# Nighttime lights, urban features, household poverty, depression, and obesity

**DOI:** 10.1007/s12144-022-02754-3

**Published:** 2022-02-16

**Authors:** Yi-An Liao, Liliana Garcia-Mondragon, Deniz Konac, Xiaoxuan Liu, Alex Ing, Ran Goldblatt, Le Yu, Edward D. Barker

**Affiliations:** 1 Social, Genetic and Developmental Psychiatry Centre, Institute of Psychiatry, Psychology and Neuroscience, King’s College London, London, SE5 8AF UK; 2 International Max Planck Research School for Translational Psychiatry (IMPRS-TP), Munich, 80804 Germany; 3 Max Planck Institute of Psychiatry, Munich, 80804 Germany; 4 Department of Psychology, Institute of Psychiatry, Psychology & Neuroscience, King’s College London, 16 De Crespigny Park, London, SE5 8AF UK; 5 Department of Psychology, Adana Alparslan Turkes Science and Technology University, Adana, Turkey; 6 Department of Earth System Science, Ministry of Education Key Laboratory for Earth System Modeling, Institute for Global Change Studies, Tsinghua University, Beijing, 100084 People’s Republic of China; 7 Aerospace Information Research Institute, Chinese Academy of Sciences, Beijing, 100190 People’s Republic of China; 8 European Molecular Biology Laboratory, Meyerhofstraße 1, Heidelberg, 69117 Germany; 9 New Light Technologies Inc., Washington, DC 20005 USA; 10 Ministry of Education Ecological Field Station for East Asian Migratory Birds, Beijing, 100084 People’s Republic of China

**Keywords:** Nighttime Light Emission, Urban features, Depression, Obesity, Network analysis

## Abstract

Nighttime Light Emission (NLE) is associated with diminished mental and physical health. The present study examines how NLE and associated urban features (e.g., air pollution, low green space) impact mental and physical wellbeing. We included 200,393 UK Biobank Cohort participants with complete data. The study was carried out in two steps. In Step1, we assessed the relationship between NLE, deprivation, pollution, green space, household poverty and mental and physical symptoms. In Step2, we examined the role of NLE on environment-symptom networks. We stratified participants into high and low NLE and used gaussian graphical model to identify nodes which bridged urban features and mental and physical health problems. We then compared the global strength of these networks in high vs low NLE. We found that higher NLE associated with higher air pollution, less green space, higher economic and neighborhood deprivation, higher household poverty and higher depressed mood, higher tiredness/lethargy and obesity (R_training_mean_ = 0.2624, *P*_training_mean_ < .001; R_test_mean_ = 0.2619, *P*_test_mean_ < .001). We also found that the interaction between environmental risk factors and mental, physical problems (overall network connectivity) was higher in the high NLE network than in the low NLE network (t = 0.7896, *P* < .001). In areas with high NLE, economic deprivation, household poverty and waist circumference acted as bridge factors between the key urban features and mental health symptoms. In conclusion, NLE, urban features, household poverty and mental and physical symptoms are all interrelated. In areas with high NLE, urban features associate with mental and physical health problems at a greater magnitude than in areas with low NLE.

**Supplementary Information:**

The online version contains supplementary material available at 10.1007/s12144-022-02754-3.

## Introduction

Fifty-five percent of the world’s population lives in urban areas and this is estimated to increase to 65% by 2050 (Heilig, 2012). Although urban areas provide beneficial possibilities, such as employment and access to public services, urban lifestyle is also associated with higher mental and physical health problems (Nieuwenhuijsen, 2016). With regard to mental health problems, the assumption is that features of the urban areas contribute to psychological distress.

### Urban Lifestyle and Mental Health

Urban areas are characterized by heavy traffic and hence, a higher emission of air pollutants (e.g., NO_2_, NOx and PM_2.5_), that is associated with 18–39% increased odds of common mental health problems (e.g., fatigue, sleep problems, irritability, depression, anxiety) (Bakolis et al., 2020). Urban areas are also associated with less green space, which is a risk factor for depression symptoms (Sarkar et al., 2018). Exposure to green space has been shown to have stress-relieving effects especially within urban areas. Theories posit that these stress-relieving effects are a result of our histories being human beings evolved primarily within nature, and thus are suggested to be inherently biophilic (attracted to nature; Hartig et al., 2003; Herzog et al., 2003; Kaplan & Kaplan, 1989; Ulrich et al., 1991; Ulrich, 1983). Indeed, more time spent in green space is associated with better mental health (van den Berg et al., 2016). This has relevance during the COVID-19 pandemic, in which low exposure to nature (i.e. green space) acted as a barrier to psychological resilience, whereas high exposure to nature facilitated psychological resilience (Tanhan, 2020).

### Urban Lifestyle and Physical Health

The associations between urban lifestyle and physical health (e.g., obesity and physical activities) in urban areas are more complex. On the one hand, proximity to fast food within densely populated areas associates with higher obesity (Bodor et al., 2010). On the other hand, services and shops within short distances encourage travelling by walking and cycling, and leads to more energy expenditure (i.e. physical exercise, Saelens et al., 2003).

### Nighttime Light Emission and Mental, Physical Health

An important urban feature, gaining recent attention, that relates to both mental and physical health, is nighttime light emission (NLE), which can be detected by remotely sensed imagery. NLE may disturb the day/night circadian rhythm, via the suprachiasmatic nucleus, which receives input from photosensitive retinal ganglion cells, and which projects axons to emotion-related brain regions, e.g., prefrontal cortex (Sylvester et al., 2002). Indeed, NLE is found to be associated with higher anxiety, depressive symptoms and suicidal ideation or attempt (Min & Min, 2018; Paksarian et al., 2020). Of interest, NLE also positively associates with obesity, perhaps through disrupted regulation of the diurnal metabolic rhythm of insulin and glucagon (Albreiki et al., 2017). A population-based survey has indeed reported an association between NLE and obesity (Koo et al., 2016).

Nonetheless, it is important to note that NLE co-occurs with other urban features that are also linked to mental and physical health problems. For example, higher NLE associates with higher air pollution due to vehicle exhaust emissions (e.g., PM_2.5_) (Helbich et al., 2020). Previous studies have also shown that vehicle exhaust emissions correlate with higher depression and rates of suicide, which may be due to neuroinflammation process triggered by the fine particles (Braithwaite et al., 2019).

### Purpose of the Paper

Given the intertwining of NLE and urban features and their combined associations with mental and physical health problems, we sought to examine these variables within an integrative analytic framework. In such a framework, our guiding framework was syndemic theory, in which two or more risk factors act synergically and contribute to the diminished health condition (Mendenhall et al., 2017). We further broadened this theory by hypothesizing that two and more health problems will be clustered in an environment with multiple risk factors.

We thus examined the simultaneous relationships between NLE, pollution, green space, economic and neighborhood deprivation, depression and anxiety symptoms, sleep patterns, BMI, waist circumference and physical activity. The first aim of this study was to identify whether these variables simultaneously associate with each other. The second aim was to assess whether the key urban features are associated with mental and physical health problems to a greater magnitude at high versus low levels of NLE.

## Materials and Methods

### Analytical Sample

#### UK Biobank Project

UK Biobank (UKBB) is a population-based cohort including more than 500,000 adults (5.5% response rate) who live in the United Kingdom (UK). The data collected at baseline (2006–2010) was used in this study. To minimize the confounding effects from the potential genetic-predisposed, ethnicity-specific circadian rhythms (Eastman et al., 2015; Egan et al., 2017; Malone et al., 2016), we selected for inclusion only white participants who had been living at the same address in England for at least three years. Participants for whom remote-sensing derived information, urban features, and individual wellbeing factors were available were included in the study, resulting in a total of 200,393 participants. UK Biobank received ethical approvals from the North West Multi-center Research Ethics Committee. The detailed cohort protocol, scientific rationale, and study design are described elsewhere (Sudlow et al., 2015).

#### Geo-Position Data Acquisition

The location information for each participant is based on the postcode associated with their home address, which was converted to east and north coordinates in the Ordnance Survey (OSGB) reference, rounded to the nearest 1 km and then converted into geographic longitude and latitude.

#### NLE Data Acquisition

**Fig. 1:**
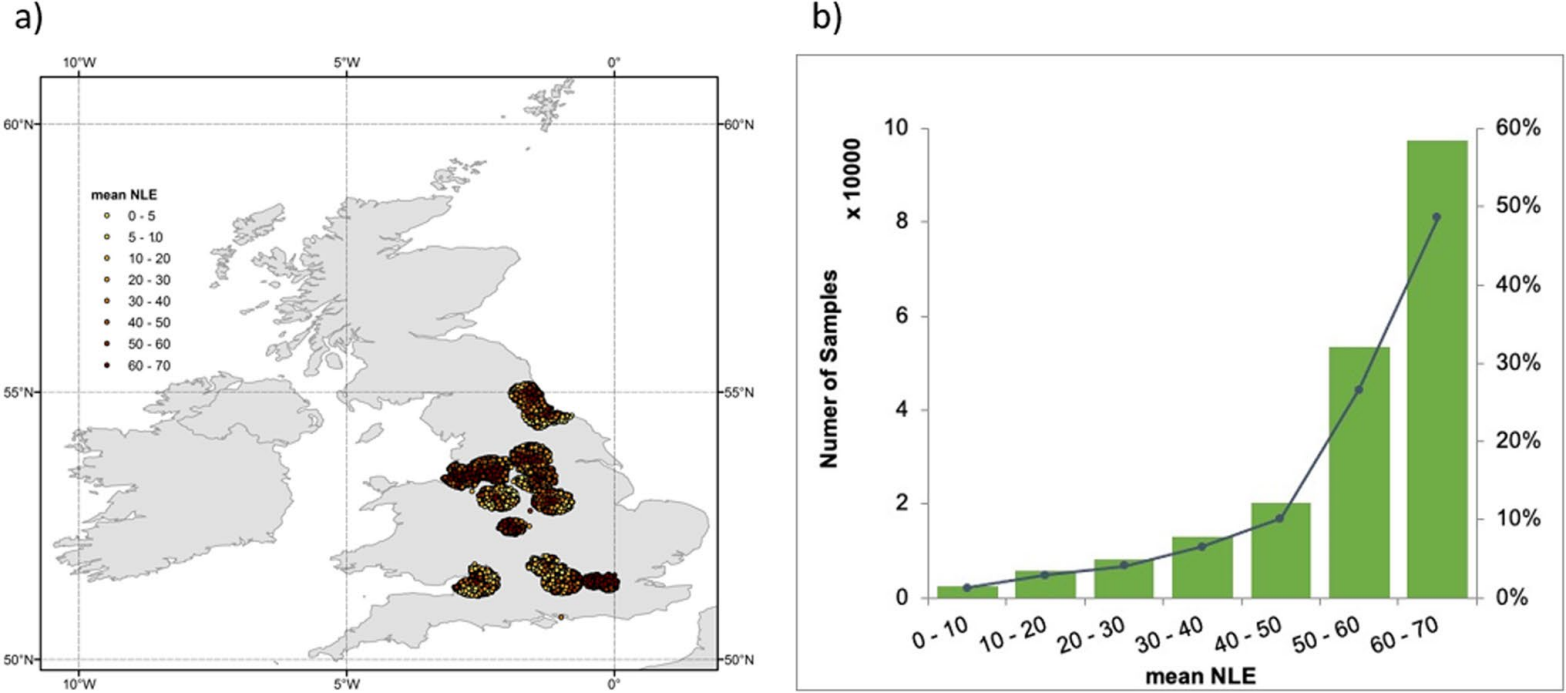
The NLE used in this study is the five-year mean NLE value (2006–2010). a) Geographical distribution of the samples (n = 200,393) in this study. b) The histogram of the samples (n = 200,393) based on the five-year mean NLE value

NLE data was collected by sensors onboard DMSP/OLS (The Defense Meteorological Program/Operational Line-Scan System) NTL (Version 4) provided by the Earth Observation Group (EOG) of the Payne Institute for Public Policy, Colorado School of Mines (Baugh et al., 2010; Elvidge et al., 1997), which includes annual NLE composites from 1992 to 2013. OLS data has been leveraged in the literature, for example, to monitor human settlements, population growth, socio-economic activity, energy consumption and the ecological footprint of human activity (Huang et al., 2014; Small et al., 2005). With 14 orbits per day, each OLS captures every location on Earth every 24 h, with a swath width of ~3000 km and a spatial resolution of 30 arc sec (1 km). The digital number (DN) of the calibrated light intensity of each OLS pixel ranges from 0 to 63. DMSP/OLS data was extracted from Google Earth Engine (GEE) (https://earthengine.google.com/), a cloud-based platform for planetary-scale analysis. Since we were interested in the effect of long-term NLE exposure on individual wellbeing, we calculated per-pixel mean value of NLE throughout the baseline period (a mean value was calculated for each pixel across five annual composites collected by F16 satellite (for 2006, 2007, 2008 and 2009) and F18 satellite (for 2010). The distribution of the NLE samples is illustrated in Fig.1.

#### Categories for Urban Features

Urban feature measures are derived from participants’ living addresses and available from UKBUMP (Sarkar et al., 2015). A set of 275 urban feature measures was selected, which included measures of air and sound pollution, accessibility to green spaces, indexes for economic deprivation, indexes for neighborhood deprivation, different measures for land-use density (e.g., health care, factory, public service), and slope. To reduce the total set of variables, we first grouped variables of like into categories. For example, “close to major road”, “inverse distance to the nearest major road” were grouped into “traffic”; “nitrogen dioxide”, “nitrogen oxides”, “PM2.5″, and “PM10” were grouped into “air pollution”. The complete set of grouped variables and their constituent measures is presented in Table S1 in the Supplementary Online Content. After z-normalizing these variables, a PCA was carried out for each group. Only measures that had a positive loading equal to or greater than 0.3 in the first component were retained. The first component was used as the score for each category.

#### Individual Wellbeing Factors

The individual wellbeing factors included three main domains:

(1) Mental wellbeing (nine measures of depression and anxiety symptoms); (2) Physical wellbeing (four measures of obesity, three measures of physical activity and six measures of sleep pattern); (3) Economic wellbeing (one measure of household poverty). All measures were extracted from the UKBB baseline. The complete description of these individual wellbeing factors is provided in Table S2 in the Supplementary Online Content.

Depression and anxiety symptoms. Depression and anxiety symptoms were extracted from the Mental Health category in UKBB. Depression symptoms included: “Frequency of depressed mood in last two weeks”, “Frequency of unenthusiasm/disinterest in last two weeks”, “Frequency of tenseness/restlessness in last two weeks”, and “Frequency of tiredness/lethargy in last two weeks”. Anxiety symptoms included “Irritability”, “Nervous feelings”, “Worrier / anxious feelings”, “Tense/‘highly strung’”, and “Worry too long after embarrassment”.

##### Obesity Measures

The obesity measures included body weight (Kg), body mass index (Kg/m^2^), waist circumference, and body fat percentage (bioelectric impedance). Waist circumference was assessed by the UKBB research staff with a measuring tape while participants stood upright with their arms crossed on their chest. Body fat percentage was assessed using Tanita BC418MA body composition analyzer, ranging from 1% to 75% in 0.1% increments.

##### Physical Activity

Participants were asked how many days in a typical week they engaged in vigorous physical activity, moderate physical activity, or walking for more than 10 min. Vigorous physical activity was described as activities that “make people sweat or breathe hard”, e.g., “fast cycling, aerobics, heavy lifting”. Moderate physical activity was described as “carrying light loads, cycling at a normal pace”.

##### Sleep Pattern

“Sleeplessness/insomnia” was defined based on the difficulty of falling asleep and continuing sleeping. “Getting-up in the morning” was based on the self-reported difficulty of getting up in the morning. Participants were asked whether they took a nap and how likely it was that they fell asleep unintentionally during the daytime. “Snoring” was assessed based on the self-reported complaints from partners or friends, and “Sleep duration” was defined as the number of hours participants slept within a 24 h period.

##### Household Poverty

Economic wellbeing at the individual level was represented by household poverty. According to the average total yearly household income before tax, participants were categorized into five income categories (less than £18,000, £18,000 to £29,999, £30,000 to £51,999, £52,000 to £100,000, greater than £100,000). The factor for household poverty was inversely coded based on these income categories.

##### Population Density

Population density was based on the attribute “Home area population density - urban or rural”, which combines the home postcode of each participant with data derived from the 2001 census from the Office of National Statistics, utilizing the Geoconvert tool from Census Dissemination Unit. In UKBB, population density has five categories: Urban, Town and Fringe, Village, Hamlet, and Dwelling.

##### Covariates

NLE was controlled for age, gender, and population density. Categories for urban features were controlled for age, gender, and population density. Individual wellbeing factors were controlled for age, gender, population density, and assessment centers.

### Statistical Methods

- Step1. Linking NLE to urban features and individual wellbeing factors by using msCCA

In Step1, we did a multiple sparse canonical correlation analysis (msCCA) with an in-sample validation to establish the relationship between NLE, urban feature categories, and individual wellbeing factors. This analysis was conducted in Matlab (R2018b). Canonical correlation analysis (CCA) is a statistical method used to identify multivariate linear associations between two sets of variables, often referred to as data ‘views’. The results of CCA can be hard to interpret since all variables are typically associated with non-zero weights. To enhance interpretability, *L*_*1*_ penalty is introduced to CCA, termed sCCA (Witten & Tibshirani, 2009) and negligible non-zeros loadings are forced to take an exact zero value. An extension of sCCA, multiple sparse canonical correlation analysis (msCCA) was designed to accommodate data with more than two views while still imposing sparsity on each view to enhance interpretability (Ing et al., 2019; Tenenhaus et al., 2014).

**Fig. 2:**
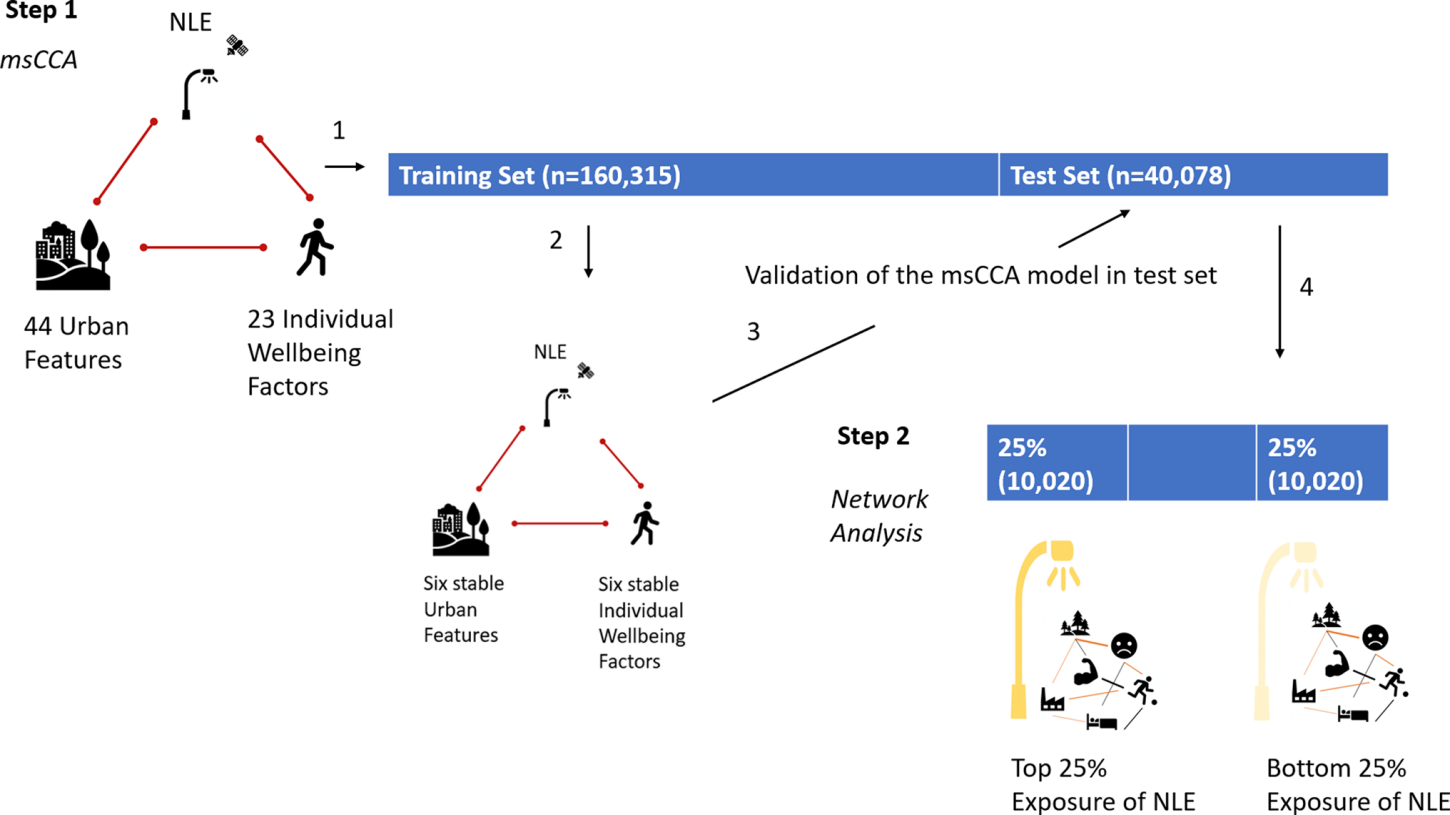
Conceptual framework of the analysis. 1. msCCA was conducted on NLE, 44 urban features and 23 individual wellbeing factors in the training set (n = 160,315). 2. In the training set, stability selection with random sparsity was conducted 1000 times. Six urban features and six individual wellbeing factors had non-zero loadings above 75% of the trials, and were considered as stable variables. The correlations between NLE, six stable Urban Features and six stable Individual Wellbeing Factors were computed. 3. The model was validated in the test set (n = 40,078), to ensure no overfitting had occurred. 4. Top 25% NLE samples and bottom 25% NLE samples were extracted from the test set for network analysis, to assess the difference between symptom network in high NLE and symptom network in low NLE

In this study, a hold-out design was used to obtain an unbiased estimate of model performance. Participants were randomly divided into a training set (80%, n = 160,315) and a test set (20%, n = 40,078). A stability selection process with 50% subsampling and random sparsity for 1000 times was conducted in the training set to identify stable urban features categories and individual wellbeing factors (Fig.2). Urban features and individual wellbeing factors appeared with non-zero loadings in more than 75% of the stability selection subsampling trials was considered stable (Meinshausen & Bühlmann, 2010). Then, msCCA was applied to the whole training dataset using the stable predictors without imposing any sparsity. This step is for ascertaining the correlation coefficient value and the loadings of the stable urban feature categories and individual wellbeing factors. For validation, the loadings generated from the training set were applied to the test set to generate the correlation coefficient of the test set. To ascertain the significance level, permutation tests for 1000 times were conducted for the results of the training set and the test set.

- Step2. Using network analysis to identify the variables bridging environment and mental-physical symptoms.

Using network analysis, in Step2 we examined how the environment and mental-physical symptoms interact with each other at high and low levels of NLE exposure. To avoid double-dipping, Step2 was only conducted with the test set. Participants in the test set (n = 40,078) who were exposed to the NLE greater than 75th percentile NLE value (top 25%, n = 10,020) and the ones exposed to the NLE lower than 25th percentile NLE values (the bottom 25%, n = 10,020) were selected for network analysis (Fig.2).

The identified stable urban feature categories and individual wellbeing factors from Step1 were regrouped into two communities for network analysis: environment and mental-physical symptoms. The environment community indicators included: air pollution, green space, economic deprivation, neighborhood deprivation, distance to education, distance to public services, and household poverty. Mental-physical symptoms included: depressed mood, unenthusiasm/disinterest, tiredness/lethargy, waist circumference, and nap during day.

Gaussian Graphical Model (GGM) was estimated for each participant group by using the ggmModSelect function implemented in the R package, *qgraph* (Epskamp et al., 2012). Being a partial correlation network, every edge between two nodes in a GGM network indicates a conditional independence association, namely, an association after controlling for all other nodes in the network (Schellekens et al., 2020). For evaluating the importance of each node (i.e. centrality), we used the R package *networktools* to compute the 1-step bridge expected influence, which is the sum of all edge weights linking the given node to all nodes in other predefined communities (Jones et al., 2021; Opsahl et al., 2010). Keeping the original signs of edge weights, the 1-step bridge expected influence can be regarded as a cumulative influence on the whole network activation (Robinaugh et al., 2016). Nodes with the top 20% of the value of the 1-step bridge expected influence were considered as bridge nodes in the network. The stability of the networks was estimated by case-dropping bootstrapping (n boots = 1000) using the R package *bootnet* (Epskamp et al., 2018). To statistically assess the difference between networks at the high and low levels of NLE exposure, we employed the permutation-based Network Comparison Test (NCT) (van Borkulo et al., 2016). This test evaluates the difference in network structure and global strength. The latter is defined as the weighted absolute sum of all the edges in a network, estimating the overall connectivity among nodes within a given network. All network analysis was conducted in RStudio (R_Core_Team, 2013).

## Results

### Descriptive Statistics

The sample consisted of 200,393 participants (49.51% male). Mean age, mean NLE exposure, information on qualifications, partnership, and household income are presented in Table 1.

**Table 1:** Demographics of the data sets

	Whole data	Training set	Test set	High NLE	Low NLE
Number	200,393	160,315	40,078	10,020	10,020
Gender (male) No.(%)	99,218(49.51)	79,294(49.46)	19,924(49.71)	5217(52.07)	5026(50.16)
Age (SD)	56.46(7.94)	56.47(7.93)	56.41(7.96)	57.69(7.53)	56.61(7.88)
Mean NLE(SD)	53.46(13.00)	53.48(13.00)	53.41(12.99)	59.79(7.03)	36.90(13.70)
Qualifications No.(%)					
College/University	69,983(34.92)	55,865(34.85)	14,118(35.23)	3931(39.23)	3566(35.59)
A level/AS levels	24,191(12.07)	19,350(12.07)	4841(12.08)	1074(10.72)	1362(13.59)
O level/GCSEs	45,475(22.69)	36,433(22.73)	9042(22.56)	1927(19.23)	2326(23.21)
CSEs	10,897(5.44)	8712(5.43)	2185(5.45)	453(4.52)	504(5.03)
NVQ/HND/HNC	13,480(6.73)	10,792(6.73)	2688(6.71)	640(6.39)	675(6.74)
Other qualifications	9999(4.99)	8047(5.02)	1952(4.87)	469(4.68)	513(5.12)
None of above	26,368(13.16)	21,116(13.17)	5252(13.10)	1526(15.23)	1074(10.72)
Partnership No.(%)					
Living alone	29,050(14.50)	23,241(14.50)	5809(14.49)	1898(18.94)	1128(11.26)
Living with a partner	158,665(79.18)	126,984(79.21)	31,681(79.05)	7421(74.06)	8366(83.49)
Household income No.(%)					
>£100,000	12,124(6.05)	9647(6.02)	2477(6.18)	858(8.56)	596(5.95)
£52,000 to £100,000	45,841(22.88)	36,747(22.92)	9094(22.69)	2105(21.01)	2491(24.86)
£30,000 to £51,999	55,278(27.58)	44,071(27.49)	11,207(27.96)	2511(25.06)	2971(29.65)
£18,000 to £29,999	50,528(25.21)	40,436(25.22)	10,092(25.18)	2478(24.73)	2549(25.44)
< £18,000	36,622(18.28)	29,414(18.35)	7208(17.98)	2068(20.64)	1413(14.10)

- Step1. NLE is associated with key urban features and diminished individual wellbeing.

**Fig. 3:**
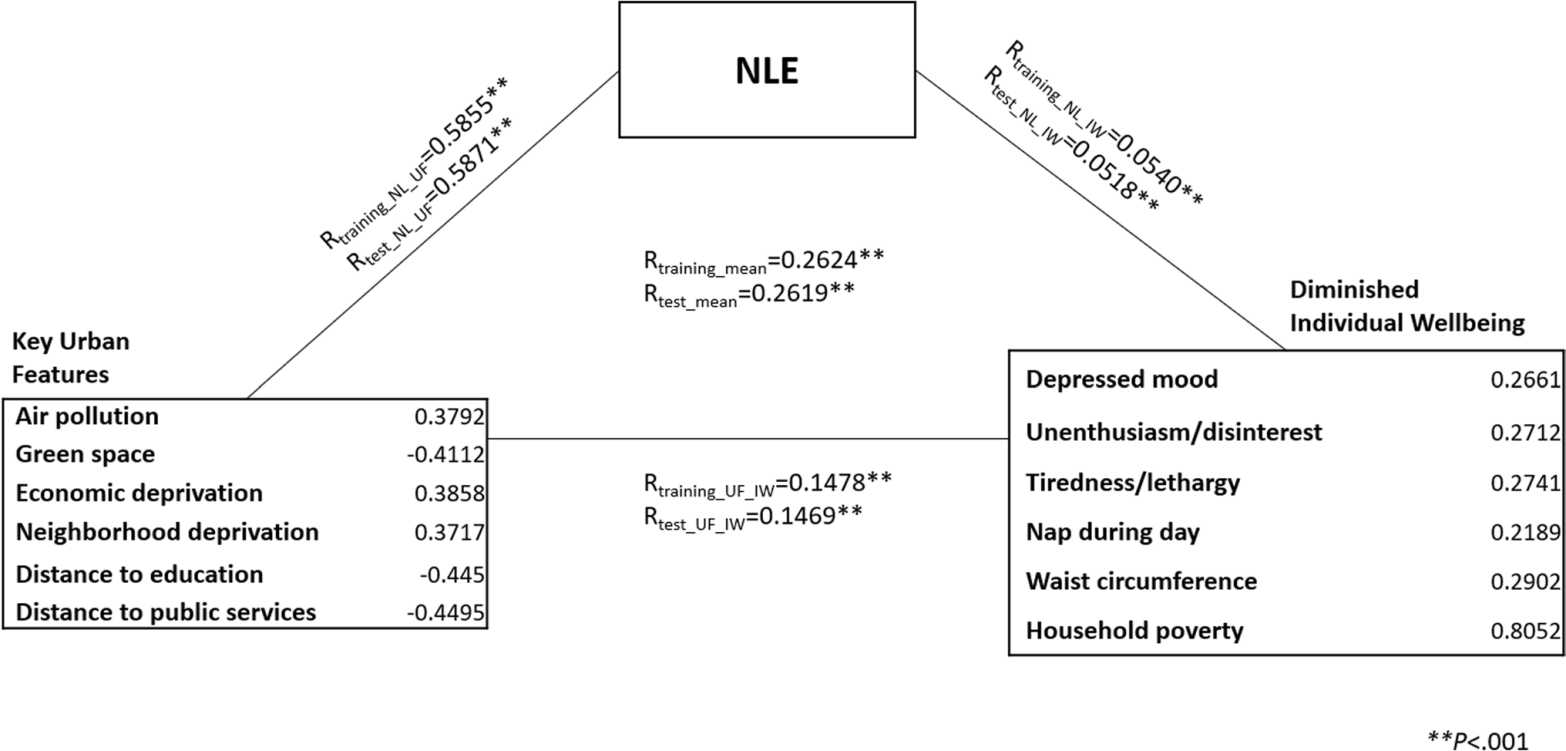
The relationship between NLE, key urban features and diminished individual wellbeing. The values of R_training_X1_X2_ and R_test_X1_X2_ correspond to the correlation coefficient between any pair of views, annotated with NL (*NL*E), UF (Key *U*rban *F*eatures) and IW (*I*ndividual *W*ellbeing). R_training_mean_ and R_test_mean_ are the means of these three correlation coefficients in training set and test set

In Step1, we found that NLE is associated with key urban features and diminished individual wellbeing (R_training_mean_ = 0.2624, *P*_training_mean_ < .001; R_test_mean_ = 0.2619, *P*_test_mean_ < .001) (Fig.3).

Among 44 urban features categories, six are key urban features: air pollution, lower green space, economic deprivation, neighborhood deprivation, shorter distance to education and public services. Six of 23 individual wellbeing factors contributed to diminished individual wellbeing. Household poverty contributed the most to the diminished individual wellbeing. Factors from the mental wellbeing included: “Frequency of depressed mood in the last two weeks”, “Frequency of unenthusiasm/disinterest in the last two weeks”, and “Frequency of tiredness/lethargy in the last two weeks”. One wellbeing factor, “Waist circumference” belonged to the obesity measures; and one wellbeing factor, “Nap during day”, belonged to the sleep pattern.

To eliminate the possibility that the overall relationship between these three views could be driven by only one correlation, we further examined the relationships between each pair of views. The correlations between NLE and diminished individual wellbeing (R_training_NL_IW_ = 0.0540, *P*_training_NL_IW_ < .001; R_test_NL_IW_ = 0.0518, *P*_test_NL_IW_ < .001), between NLE and key urban features (R_training_NL_UF_ = 0.5855, *P*_training_NL_UF_ < .001, R_test_NL_UF_ **=** 0.5871, *P*_test_NL_UF_ < .001), and the relationship between key urban features, and diminished individual wellbeing (R_training_UF_IW_ = 0.1478, *P*_training_UF_IW_ < .001; R_test_UF_IW_ = 0.1469, *P*_test_UF_IW_ < .001) were computed and validated in the test set.

- Step 2 Examination and comparison of interactions between environment and mental-physical symptoms at high and low levels of NLE

#### Bridge Nodes

**Fig. 4:**
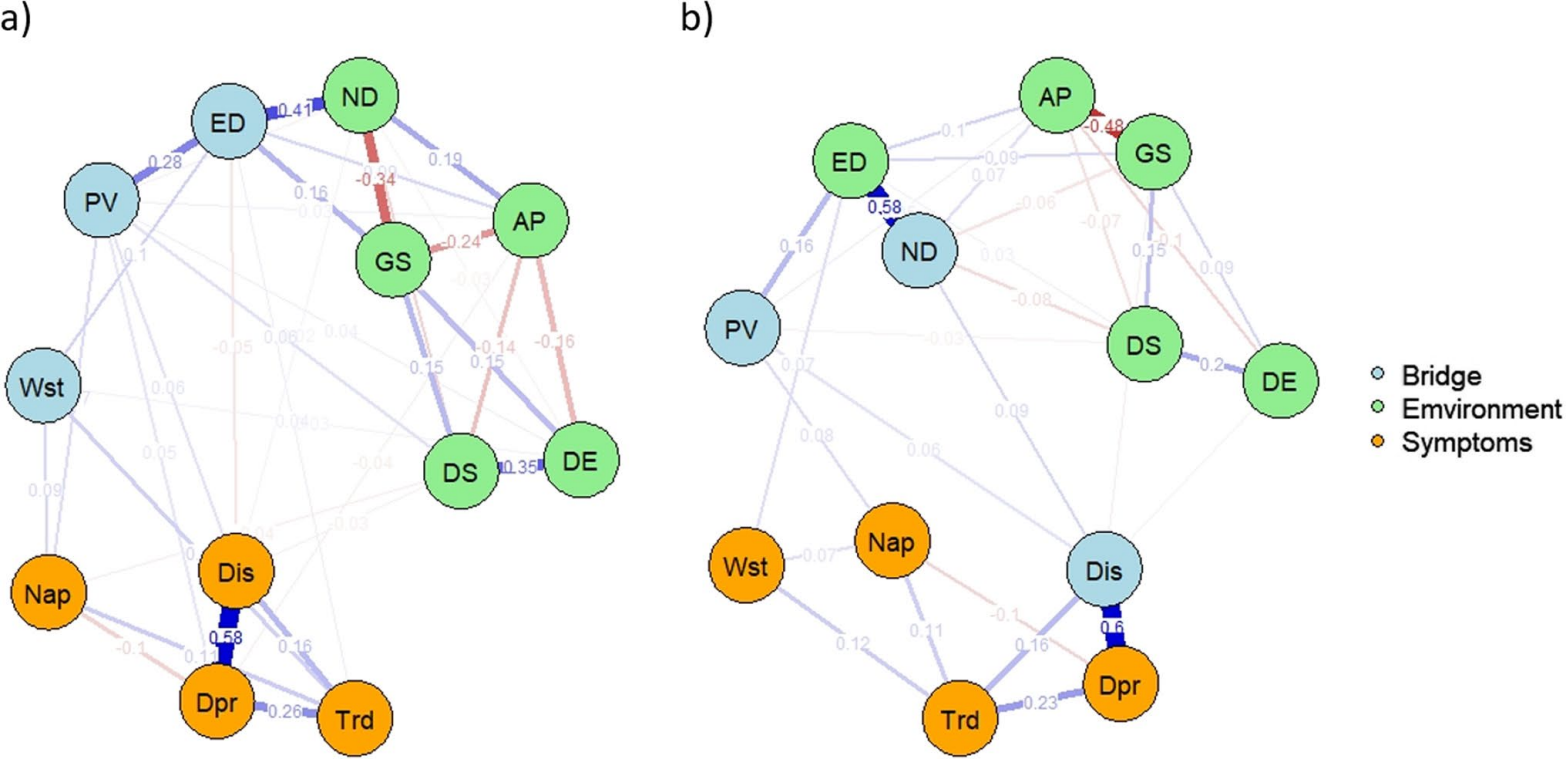
Environment-symptom networks for participants exposed to top 25% NLE (a) and bottom 25% NLE (b); Dpr: frequency of depressed mood in the last two weeks; Dis: frequency of unenthusiasm/disinterest in the last two weeks; Trd: frequency of tiredness/lethargy in the last two weeks; Nap: Nap during day; Wst: Waist circumference; AP: Air pollution; GS: Green space; ED: Economic deprivation; ND: Neighborhood deprivation; DE: Distance to education; DS: Distance to public services; PV: Household poverty

The top 25% NLE network (high NLE network) is presented in **panel a** and the bottom 25% NLE network (low NLE network) is presented in **panel b** in Fig.4. The top 20% scoring nodes on 1-step bridge expected influence were colored in blue and labeled as “bridge” in the networks.

There are more edges in the high NLE network than in the low NLE network. In the high NLE network, environment nodes are connected more densely with the mental-physical nodes. In the low NLE network, on the contrary, there are fewer edges between environment nodes and mental-physical nodes.

In both high and low NLE networks, household poverty was identified as a bridge node bridging environment and mental-physical symptoms.

In the high NLE exposure network, two other nodes, economic deprivation and waist circumference, bridged environment and mental-physical symptoms. In the low NLE exposure network, neighborhood deprivation and unenthusiasm/disinterest were identified as bridge nodes between environment and mental-physical symptoms.

#### Network Structure and Node Interconnection

To quantitatively assess the difference between high and low NLE networks, we used the network comparison test to compare the structure and the global strength between these two networks. The structure between high and low NLE networks significantly differed (invariance test: t = 0.2749, *P* < .001). Moreover, we found that the global strength was greater in the high NLE network than in the low NLE network (t = 0.7896, *P* < .001), meaning that there was a greater interconnection between nodes in the high NLE network.

## Discussion

To the best of our knowledge, this is the first study to simultaneously examine the relationships between NLE, urban features and mental and physical health. In the first step of the analysis, we found that NLE and key urban features are associated with higher mental and physical health problems. In the second step, we found that within geographic areas characterized by high NLE vs low NLE, in general, the key urban features formed a tighter network with the mental and physical health problems. More specifically, within these comparative network models, in areas with high NLE, economic deprivation, household poverty and waist circumference acted as bridge factors between the key urban features (e.g., green space, air pollution and neighborhood deprivation) and mental health symptoms (e.g., disinterest, feeling depressed). In contrast, in areas with low NLE, neighborhood deprivation, household poverty, and unenthusiasm/disinterest bridged the key urban features (e.g., economic deprivation) and mental health symptoms (e.g., disinterest, feeling depressed), but of interest, not with waist circumference.

### Simultaneous Association between NLE, Urban Features and Poor Mental, Physical Health

Our findings add to existing knowledge in three ways. First, as stated, we simultaneously examined NLE, urban features and mental and physical health, and indeed found that higher NLE associated with higher air pollution, lower green space and higher economic deprivation, as well as depressed mood, disinterest, tiredness and obesity. Previous studies have suggested that the correlation between NLE and mental, physical symptoms can be explained by the biological role of light in circadian rhythm (Paksarian et al., 2020). Animal studies have shown that visual pathways transmitting light information also project axons to the emotion control center; and the downstream of these pathways are involved in metabolism, generating circadian oscillation of hormones, including insulin, leptin (Fernandez et al., 2018; Hattar et al., 2006; Kalsbeek et al., 2001). This neuroanatomical framework can explain how NLE is associated with depressive symptoms.

### Syndemic Clustering of Depression, Obesity, Poverty in High NLE Areas

Our second set of novel findings related to the mechanisms that may contribute to multimorbidity, or the association(s) between mental and physical health problems. A traditional approach to multimorbidity, especially when it comes to the mental and physical health problems that co-occur (e.g., depression and weight gain), often ignores the complex social and geographic contexts in which the multimorbidity is more likely to arise (Mendenhall et al., 2017; Mustanski et al., 2007). A syndemic point of view characterizes comorbidities as synergies between individuals and their social contexts (e.g., exposure to structural inequalities). Here, health conditions are often clustered within a population in certain geographic contexts with higher risk exposures increasing vulnerabilities for disease (Mendenhall et al., 2017). Following this line of thought, our findings demonstrate that depression, obesity and household poverty are syndemic in areas characterized by high NLE, high air pollution, less accessibility to green spaces, and economic, neighborhood deprivation. A recent study further indicated that urban residents complained about being forced to stay away from green spaces, and that it made them feel more stressed (Tanhan, 2020).

### Higher Urban-Symptom Interaction in High NLE Areas

Our third novel finding relates to the network models, where we compared the strength of connections between the key urban features and the symptoms of mental and physical health problems, at high and low levels of NLE. Network analysis allows one to evaluate if these variables form a strongly-interconnected network, where the key urban features and mental and physical health symptoms can influence, or activate each other (Hevey, 2018). Here, we found that the global strength for the overall network was higher in high NLE areas than in low NLE areas. We also examined bridge nodes, i.e., nodes that connect the key urban features to the mental and physical health symptoms. Of interest, obesity was only identified at high NLE. More specifically, economic deprivation, household poverty and waist circumference acted as bridge nodes between the key urban features (e.g., air pollution, green space, and neighborhood deprivation) and the mental health symptoms (e.g., feeling depressed, unenthusiasm/disinterest) at high NLE. Of interest, visceral fat, in particular, has a strong association with depression (Vogelzangs et al., 2008). Waist circumference is a well-established measure for central obesity, which reflects the accumulation of visceral fat. Excess visceral fat can lead to inflammation, metabolic and cardiovascular diseases, and depression (Després, 2007; Fontana et al., 2007; Zhao et al., 2011). Possible biological pathways explaining the co-occurrence of obesity and depressed mood, as reported here, include inflammatory activation, disturbed hypothalamic–pituitary–adrenal (HPA) axis and altered insulin metabolism (Milaneschi et al., 2019).

In contrast, in areas with low NLE, neighborhood deprivation and unenthusiasm/disinterest bridged the key urban features (e.g., economic deprivation) and mental health symptoms (e.g., feeling depressed). But it is important to note that economic deprivation was a key risk factor for both low and high NLE areas. In the present study, neighborhood deprivation included risk in the built environment (poor housing conditions) as well as social environment (criminal victimization). Although the causality between poor housing and diminished mental health is under debate, a UK study has shown decreased Housing Benefits resulted in higher depressive symptoms for people living within poverty (Reeves et al., 2016). These results indicate that deprived living conditions can lead to the deterioration of mental health. In addition, victimization is often clustered at a micro-geographic level, termed criminal hot spots (Weisburd, 2015). Compared with non-hot spots, hot spots are characterized not only by poverty but also by a higher prevalence of depression (Weisburd & White, 2019). Our results support the notion that victimization hot spots may also be linked with the development of mental-physical symptoms, in particular, in low NLE areas.

## Limitations

The findings in this study should be interpreted in light of numerous limitations. Firstly, the causality between NLE and mental and physical health problems is still controversial (McIsaac et al., 2021). Although it is unlikely that mental and/or physical health problems can cause higher NLE, pollution, green space or economic deprivation, individuals with certain heritable traits may prefer to live in areas with higher NLE and key urban features. Secondly, due to the collection time range of our sample (2006–2010), we were not able to use the VIIRS-DNB dataset (since 2012), which have 7 times better linear resolution compared to DMSP/OLS (Elvidge et al., 2013). An atlas of artificial sky luminance has been created based on VIIRS-DNB (Falchi et al., 2016). Future studies using more recent cohorts should employ the VIIRS-DNB dataset to better delineate the epidemiological relationship between NLE and mental, physical health problems. Thirdly, it should be noted that this study is based on the mean NLE level throughout the baseline period, as well as a cross-sectional slice of exposures to urban features and experiences of mental and physical health problems. In other words, this study does not show how trends in urbanization (i.e. the change in urban features), or change in NLE level can impact these mental and physical health problems. Fourthly, this study is based on the UK Biobank, and the results cannot be easily transferred to developing countries. Lastly, because in this study we did not include clinical data, the translation of its findings to clinical contexts remains unknown.

## Conclusions

In this study, we investigated the relationship between NLE, urban features and mental and physical health. We demonstrated that the strength of the relationship between these variables is stronger in areas with high NLE than in areas with low NLE. Further studies should clarify the causality of the relationship between NLE, urban features and mental and physical health problems. Cohorts from developing countries should also be included in follow-up studies, to gain a more comprehensive view of how urbanicity is associated with an individual’s ability to flourish and thrive across the life course.

## Implication

The present study provides a new approach to identify the environmental characteristics in which residents are likely to develop physical and mental health problems. This type of approach may be useful for health workers and policy decision-makers. In addition, urban designers can also take these findings into consideration for improving urban designs that will benefit physical and mental health. We also note that the satellite and ground-level data-based in the present study could help stimulate future studies, using similar measures, examining health-environment interaction.

Of note, future studies can focus and zoom-in on the at-risk communities with the Online Photovoice methodology (Tanhan, 2020; Tanhan & Strack, 2020). The traditional photovoice methodology encouraged participants to document their environmental exposures and experiences through taking photographs. The online version of photovoice allows researchers to reach out the marginalized participants more efficiently. Furthermore, the zoom-in approach, in which the environment is further characterized by satellite measurement and ground-level census data, acts as an excellent data resource in addition to the Online Photovoice documentation.

## Supplementary Information

ESM 1 (DOCX 34 kb)

## Data Availability

The behavioural data and the spatial environmental data (UKBUMP) that support the findings of this study are available from UK Biobank. The nightlight data (DMSP/OLS data) is available from Google Earth Engine.
