# Supplementary material for: Nighttime lights, urban features, household poverty, depression, and obesity
Source: Curr Psychol. 2022 Feb 16:1–12. Online ahead of print. doi: 10.1007/s12144-022-02754-3 (PMC8853344; doi:10.1007/s12144-022-02754-3)
Supplement: Supplementary file 1 — (DOCX 34 kb) [file 12144_2022_2754_MOESM1_ESM.docx]

**Supplementary Online Content**

**Table S1 Urban feature categories and measures**

| **Categories** | **Measures** |
| --- | --- |
| **Traffic** | Close_to_major_road |
|  | Inverse_distance_nearest_major_road |
|  | Sum_of_road_length_major_roads_within_100m |
|  | Traffic_intensity_nearest_road |
| **Air pollution** | Nitrogen_dioxide_air_pollution_2010 |
|  | Nitrogen_oxides_air_pollution_2010 |
|  | PM2_5_air_pollution_2010 |
|  | PM10_air_pollution_2010 |
| **Sound pollution** | Average_daytime_sound_level_of_noise_pollution |
|  | Average_evening_sound_level_of_noise_pollution |
|  | Average_night_time_sound_level_of_noise_pollution |
| **Green space** | Green space_percentage_buffer_1000m |
|  | Natural_environment_percentage_buffer_1000m |
| **Deprivation_income_employment_education** | IMD_score |
|  | Income_score |
|  | Employment_score |
|  | Education_skills_and_training_score |
|  | Children_Young_People_Sub_domain_score |
|  | IDACI_score |
| **Deprivation_crime_living_env_housing** | Crime_and_disorder_score |
|  | Living_environment_score |
|  | Indoors_Sub_domain_Score |
|  | Outdoors_Sub_domain_Score |
|  | Wider_Barriers_Sub_domain_Score |
| **Slope** | Slope500m_Mean |
|  | Slope500m_Maximum |
|  | Slope500m_STD |
| **Distance_education** | ND_CE01_College |
|  | ND_CE02_Childrens_Nursery_Creche |
|  | ND_CE03_Preparatory_First_Primary_Infant_Junior_M |
|  | ND_CE04_Secondary_High_School |
|  | ND_CE05_University |
| **Distance_factory** | ND_CI01_Factory_Manufacturing |
|  | ND_CI02_Mineral_Ore_Working_Quarry_Mine |
|  | ND_CI03_Workshop_Light_Industrial |
|  | ND_CI04_Warehouse_Store_Storage_Depot |
| **Distance_community** | ND_CC04_Public_Village_Hall_Other_Community_Facility |
|  | ND_CL03_Library |
|  | ND_CL07_Cinema_Conf_Exhib_Centre_Theatre_Concert_Hall |
|  | ND_ZW_Places_of_Worship |
| **Distance_healthcare** | ND_CM01_Dentist |
|  | ND_CM02_GP_Practice_Surgery_Clinic |
|  | ND_CM03_Hospital_Hospice |
| **Distance_services** | ND_CC12_Job_Centre |
|  | ND_CO01GV_Central_Government_Service |
|  | ND_CO01LG_Local_Government_Service |
|  | ND_CR01_Bank_Financial_Service |
|  | ND_CR02_Retail_Service_Agent |
|  | ND_CR02PO_Post_Office |
| **Distance_waste_and_energy** | ND_CU02_Landfill |
|  | ND_CU03_Power_Station_Energy_Production |
|  | ND_CU07_Water_Waste_Water_Sewage_Treatment_Works |
|  | ND_Recycling_Recycling |
| **Distance_transport** | ND_CT03_Parking_Park_and_Ride_Site |
|  | ND_CT08_Station_Interchange_Terminal_Halt |
| **Distance_emergency** | ND_CX01_Police_Transport_Police_Station |
|  | ND_CX02_Fire_Station |
|  | ND_CX03_Ambulance_Station |
| **Distance_food** | ND_CR06_Public_House_Bar_Night_Club |
|  | ND_CR07_Restaurant_Cafeteria |
|  | ND_CR10_Fast_Food_Outlet_Takeaway |
| **Density_agricultural** | Den_CA01_Farm_Non_Residential_Associated_Building |
|  | Den_CA02_Fishery |
|  | Den_CA03_Horticulture |
| **Density_education** | Den_CE_Education |
|  | Den_CE02_Childrens_Nursery_Creche |
|  | Den_CE03_Preparatory_First_Primary_Infant_Junior_Middle_School |
|  | Den_CE03NP_Non_State_Primary_Preparatory_School |
|  | Den_CE04_Secondary_High_School |
|  | Den_CE05_University |
| **Density_accommodation** | Den_CH01_Boarding_Guest_House_Bed_And_Breakfast_Youth_Hostel |
|  | Den_CH02_Holiday_Let_Accommodation_Short_Term_Let |
|  | Den_CH03_Hotel_Motel |
| **Density_factory** | Den_CI01_Factory_Manufacturing |
|  | Den_CI03_Workshop_Light_Industrial |
|  | Den_CI04_Warehouse_Store_Storage_Depot |
| **Density_physical_activity1** | Den_CL06_Indoor_Outdoor_Leisure_Sporting_Activity_Centre |
|  | Den_CL06CK_Cricket_Facility |
|  | Den_CL06QS_Racquet_Sports_Facility |
|  | Den_CL06WA_Water_Sports_Facility |
| **Density_physical_activity2** | Den_CL06FB_Football_Facility |
|  | Den_CL06LS_Activity_Leisure_Sports_Centre |
|  | Den_CL06RF_Rugby_Facility |
| **Density_healthcare** | Den_CM_Medical |
|  | Den_CM01_Dentist |
|  | Den_CM02_General_Practice_Surgery_Clinic |
|  | Den_CM02HC_Health_Centre |
|  | Den_CM02HL_Health_Care_Services |
|  | Den_CM05_Prof_Medical_Service_Assessment_Developm_Services |
| **Density_hospital** | Den_CM03_Hospital_Hospice |
|  | Den_CM03HI_Hospice |
|  | Den_CM03HP_Hospital |
|  | Den_CM04_Medical_Testing_Research_Laboratory |
| **Density_animal_centre** | Den_CN02_Animal_Services_Animal_Quarantining |
|  | Den_CN04_Vet_Animal_Medical_Treatment |
| **Density_food** | Den_CR06_Public_House_Bar_Nightclub |
|  | Den_CR07_Restaurant_Cafeteria |
|  | Den_CR09_Other_Licensed_Premise_Vendor |
|  | Den_CR10_Fast_Food_Outlet_Takeaway_Hot_Cold |
| **Density_emergency** | Den_CX01_Police_Transport_Police_Station |
|  | Den_CX02_Fire_Station |
|  | Den_CX03_Ambulance_Station |
| **Density_street** | Den_CZ01_Advertising_Hoarding |
|  | Den_CR11_Automated_Teller_Machine_ATM |
|  | Den_CU11_Telephone_Box |
|  | Den_Bstops_Density_of_bus_stops |
| **Density_maintained_areas** | Den_LM01_Landscaped_Roundabout |
|  | Den_LM02_Verge_Central_Reservation |
|  | Den_LM03_Maintained_Amenity_Land |
|  | Den_LM04_Maintained_Surfaced_Area |
| **Density_park** | Den_LM_Amenity_Open_areas_not_attracting_visitors |
|  | Den_LP01_Public_Park_Garden |
|  | Den_LP02_Public_Open_Space_Nature_Reserve |
|  | Den_LP03_Playground |
| **Density_unused_land** | Den_LL_Allotment |
|  | Den_LU01_Vacant_Derelict_Land |
| **Density_water** | Water_percentage_buffer_1000m |
|  | Den_LW01_Lake_Reservoir |
|  | Den_LW02_Named_Pond |
| **Density_military** | Den_M_Military |
|  | Den_MA_Army |
| **Density_residence_general** | Den_R_Residential |
|  | Den_RB_Ancillary_Building |
|  | Den_RC01_Car_Park_Space |
|  | Den_RD_Dwelling |
| **Density_residence_HMO** | Den_RH01_HMO_Parent |
|  | Den_RH02_HMO_Bedsit_Other_Non_Self_Contained_Accommodation |
|  | Den_RH03_HMO_Not_Further_Divided |
| **Density_residence_detached** | Den_RD02_Detached |
|  | Den_RD03_Semi_Detached |
|  | Den_RD04_Terraced |
|  | Den_RD06_Self_Contained_Flat_Includes_Maisonette_Apartment |
| **Density_residence_communal** | Den_RI01_Care_Nursing_Home |
|  | Den_RI02_Communal_Residence |
|  | Den_RI03_Residential_Education |
| **Density_monument** | Den_ZM01_Obelisk_Milestone_Standing_Stone |
|  | Den_ZM02_Memorial_Market_Cross |
|  | Den_ZM03_Statue |
|  | Den_ZM05_Other_Structure_Art_Display_Cascade_Fountain_Windmill |
| **Density_underground_feature** | Den_Z_Object_of_Interest |
|  | Den_ZS_Stately_Home |
|  | Den_ZU_Underground_Feature |
|  | Den_ZV_Other |
| **Density_church** | Den_ZW_Place_Of_Worship |
|  | Den_ZW99CH_Church |
|  | Den_ZW99MQ_Mosque |
|  | Den_ZW99TP_Temple |
| **Density_transport** | Den_CT_Transport |
|  | Den_CT02_Bus_Shelter |
|  | Den_CT07_Railway_Asset |
|  | Den_CT08_Station_Terminal_Halt_Bus_Coach_Railway_Station |
|  | Den_CT09_Transport_Track_Way |
|  | Den_CT10_Vehicle_Storage |
|  | Den_CT11_Transport_Related_Infrastructure |
| **Density_waste_and_energy** | Den_CC10_Recycling_Site |
|  | Den_CU_Utility |
|  | Den_CU01_Electricity_Sub_Station |
|  | Den_CU06_Telecommunication |
| **Density_community** | Den_CC04_Community_Facility_Youth_Recreat_Social_Club |
|  | Den_CC07_Church_Hall_Religious_Meeting_Place_Hall |
|  | Den_CL01_Amusements_Leisure_Pier |
|  | Den_CL03_Library |
|  | Den_CL04_Museum_Gallery |
|  | Den_CL07_Cinema_Conference_Exhib_Centre_Theatre_Concert_Hall |
|  | Den_CL10_Licensed_Private_Members_Club_Recreational_Social_Club |
| **Density_services** | Den_CC05_Public_Convenience |
|  | Den_CO01_Office_Work_studio |
|  | Den_CR01_Bank_Financial_Service |
|  | Den_CR02_Retail_Service_Agent_Post_Office |
|  | Den_CR08_Shop_Showroom_Garden_Centre |

**Table S2 Wellbeing factors**

**Depression and anxiety symptoms**

| Acronym | Name of the measure | Question | |
| --- | --- | --- | --- |
| Dpr | frequency of depressed mood in the last 2 weeks | Over the past two weeks, how often have you felt down, depressed, or hopeless? | |
| Dis | frequency of unenthusiasm/disinterest in the last 2 weeks | Over the past two weeks, how often have you had little interest or pleasure in doing things?" | |
| Ten | frequency of tenseness/restlessness in the last 2 weeks | Over the past two weeks, how often have you felt tense, fidgety, or restless?" | |
| Trd | frequency of tiredness/lethargy in the last 2 weeks | Over the past two weeks, how often have you felt tired or had little energy? | |
| Irr | irritability | | Are you an irritable person? |
| Nrv | nervous feelings | | Would you call yourself a nervous person? |
| Wor | worrier / anxious feelings | | Are you a worrier? |
| Tns | tense / 'highly strung' | | Would you call yourself tense or 'highly strung'?" |
| Emb | worry too long after the embarrassment | | Do you worry too long after an embarrassing experience? |
|  |  | |  |

**Obesity measures**

| Acronym | Name of the variable | Question |
| --- | --- | --- |
| BMI | BMI | BMI value here is constructed from height and weight measured during the initial Assessment Centre visit. |
| Wst | Waist Circumference | Waist circumference |
| Fat | Body fat percentage | Body composition estimation by impedance measurement. |
| Wei | Weight | Weight was measured by a variety of means during the initial Assessment Centre visit |

**Physical Activity**

| Acronym | Name of the variable | Question |
| --- | --- | --- |
| Vig | Number of days/week of vigorous physical activity 10+ minutes | In a typical WEEK, on how many days did you walk for at least 10 minutes at a time? (Include walking that you do at work, traveling to and from work, and for sport or leisure) |
| Mod | Number of days/week of moderate physical activity 10+ minutes | In a typical WEEK, on how many days did you do 10 minutes or more of moderate physical activities like carrying light loads, cycling at a normal pace? |
| Wit | Number of days/week walked 10+ minutes | In a typical WEEK, how many days did you do 10 minutes or more of vigorous physical activity? (These are activities that make you sweat or breathe hard such as fast cycling, aerobics, heavy lifting) |

**Sleep pattern**

| Acronym | Name of the variable | Question |
| --- | --- | --- |
| Ins | Sleeplessness / insomnia | "Do you have trouble falling asleep at night or do you wake up in the middle of the night?" |
| Gup | Getting up in the morning | On an average day, how easy do you find getting up in the morning? |
| Nap | Nap during day | Do you have a nap during the day? |
| Snr | Snoring | Does your partner or a close relative or friend complain about your snoring? |
| Doz | Daytime dozing/sleeping (narcolepsy) | How likely are you to doze off or fall asleep during the daytime when you don't mean to? (e.g. when working, reading or driving) |
| Sdu | Sleep duration | About how many hours sleep do you get in every 24 hours? (please include naps) |

**Household poverty**

| Acronym | Name of the variable | Question |
| --- | --- | --- |
| PV | Household poverty | Inversely coded variable based on yearly income before tax (income1 less than £18,000; income2: £18,000 to £29,999, income3: £30,000 to £51,999, income4: £52,000 to £100,000, income5: greater than £100,000) |
